# Complete mitochondrial genome of a subspecies of the great cormorant, *Phalacrocorax carbo hanedae* (Kuroda, 1925) (Suliformes: Phalacrocoracidae)

**DOI:** 10.1080/23802359.2022.2160671

**Published:** 2023-01-02

**Authors:** Rina Honda, Mizue Inumaru, Yukita Sato, Atsushi Sogabe

**Affiliations:** a Saitama Museum of Natural History, Nagatoro, Japan; b Department of Medical Entomology, National Institute of Infectious Diseases, Tokyo, Japan; c Department of Veterinary Medicine, Nihon University, Fujisawa, Japan; d Department of Biology, Hirosaki University, Hirosaki, Japan

**Keywords:** *Phalacrocorax carbo hanedae*, mitogenome, phylogenetic tree

## Abstract

We determined the complete mitochondrial DNA sequence of a subspecies of the great cormorant, *Phalacrocorax carbo hanedae* (Kuroda, 1925) using long PCR and primer walking methods. The mitochondrial genome was 19,020 bp in length and contained 13 protein-coding genes (PCGs), two ribosomal RNA genes, 22 transfer RNA genes, and two control regions. It is basically consistent with the characteristics of the mitochondrial genomes of other Suliformes species. Phylogenetic analysis using 12 species of Suliformes based on the sequences of 13 concatenated protein-coding genes confirmed the monophyly of *P. carbo* ssp.

## Introduction

**Figure 1.**
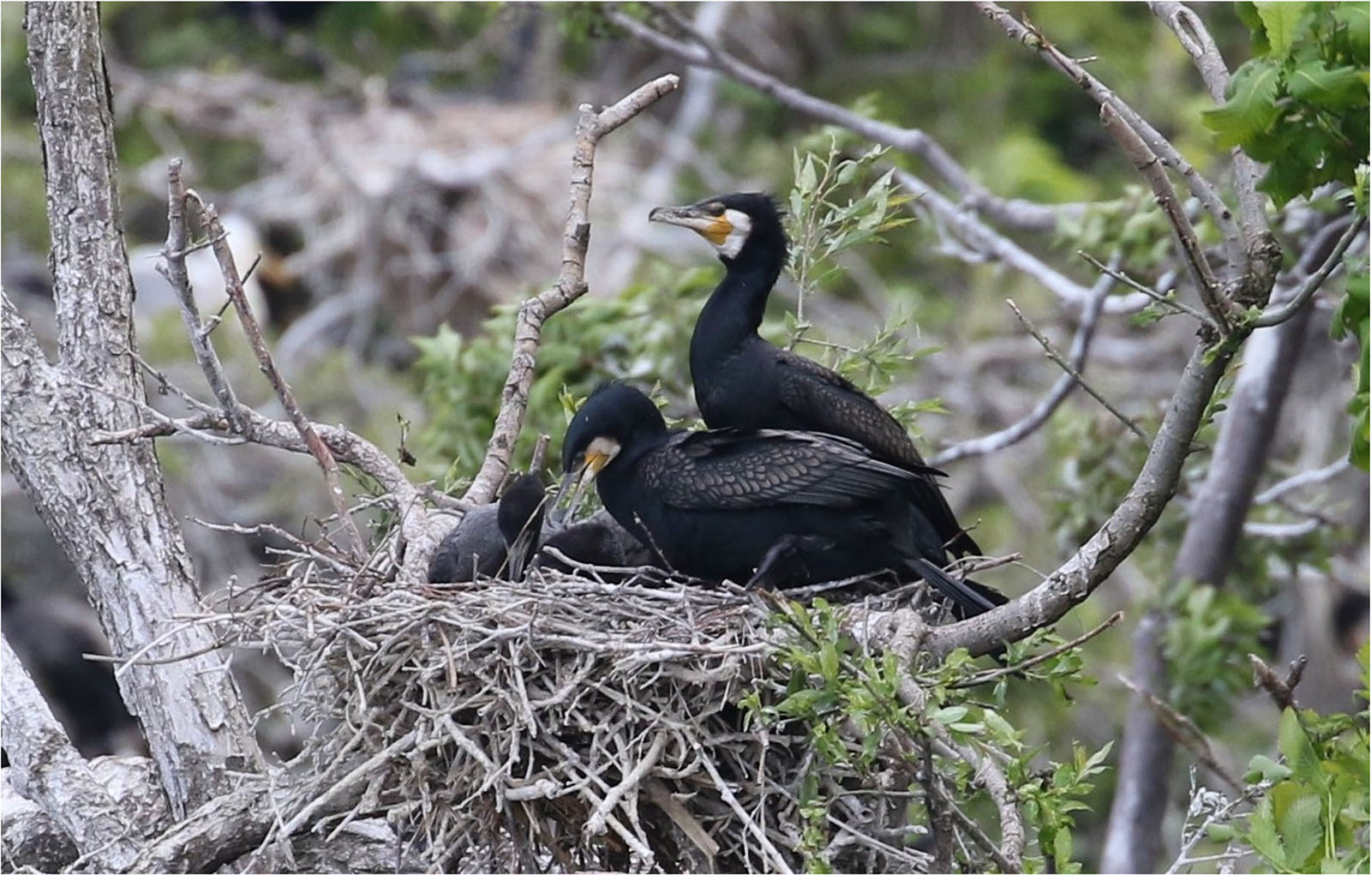
A great cormorant subspecies, *Phalacrocorax carbo hanedae*, photographed by R. Honda at Gongen-numa Pond in Aomori, Japan. Two species of cormorants, *P. carbo hanedae* and *P. capillatus*, are known to inhabit Japan, which can be distinguished by the shape of the yellow bare part at the base of their beaks. The individual in the photo can be identified as *P. carbo hanedae* because the yellow bare part at the base of the beak is not pointed at the corner of the mouth.

The great cormorant *Phalacrocorax carbo* (Linnaeus, 1758) consists of six subspecies distributed worldwide, excepting only South America and the Antarctic. The subspecies *P. carbo hanedae* (Kuroda, 1925) is endemic to Far East Asia, ranging over Taiwan, Korea, and Japan to Sakhalin (del Hoyo et al. 1992; Figure 1). The recent range expansion and population explosion of *P. carbo hanedae* in Japan has caused serious damage to freshwater fisheries. Genetic information on this subspecies is essential to promote its proper management while preserving its genetic diversity. This warrants the development of genetic markers with varying evolutionary rates to infer the genetic population structure of *P. carbo hanedae*. However, the mitogenome of this species has not previously been investigated.

## Materials and methods

A liver specimen was obtained from a dead individual of a subspecies of the great cormorant, *P. carbo hanedae,* collected by the Gyotoku Nature Conservation Club NPO in Ichikawa City, Chiba Prefecture, Japan (35°40′25.7″N, 139°55′26.0″E) on July 24, 2015. The genomic DNA was extracted using a DNeasy Tissue and Blood Kit (Qiagen, Hilden, Germany). The DNA specimen was deposited at Hirosaki University (Dr. Atsushi Sogabe, e-mail: atsushi.sogabe@hirosaki-u.ac.jp) under voucher number HUA2103162. Primer walking for the five long PCR products covering whole mitochondrial DNA was used to determine the complete mitogenome sequence (see Tables S1 and S2 for primers used in the study and Figure S1 for PCR gel image). DNA sequencing was conducted in an automated DNA analyzer ABI 3500 (Life Technologies, Carlsbad, CA). The sequence fragments were then assembled using the GeneStudio Professional version 2.2.0.0 (GeneStudio, Inc., Suwanee, GA). The assembled mitogenome sequence was annotated using MITOS web server (Bernt et al. 2013).

The phylogenetic relationships of 12 Suliformes species were reconstructed through Bayesian inference (BI) and maximum likelihood (ML) based on 13 protein-coding genes (PCGs) after selecting the best fitting model of sequence evolution using Kakusan4 (Tanabe 2011) for BI and ModelTest-NG version 0.1.6 (https://chlorobox.mpimp-golm.mpg.de/OGDraw.html; Darriba et al. 2020) for ML. ML analysis was performed using RAxML-NG version 1.0.1 (https://github.com/amkozlov/raxml-ng; Kozlov et al. 2019) with 1,000 bootstrap replicates. Bayesian phylogenetic analysis was conducted with BEAST version 1.10.4 (https://beast.community/; Suchard et al. 2018) with 100,000,000 Markov steps and 25,000,000 burn-in steps.

## Results and discussion

**Figure 2.**
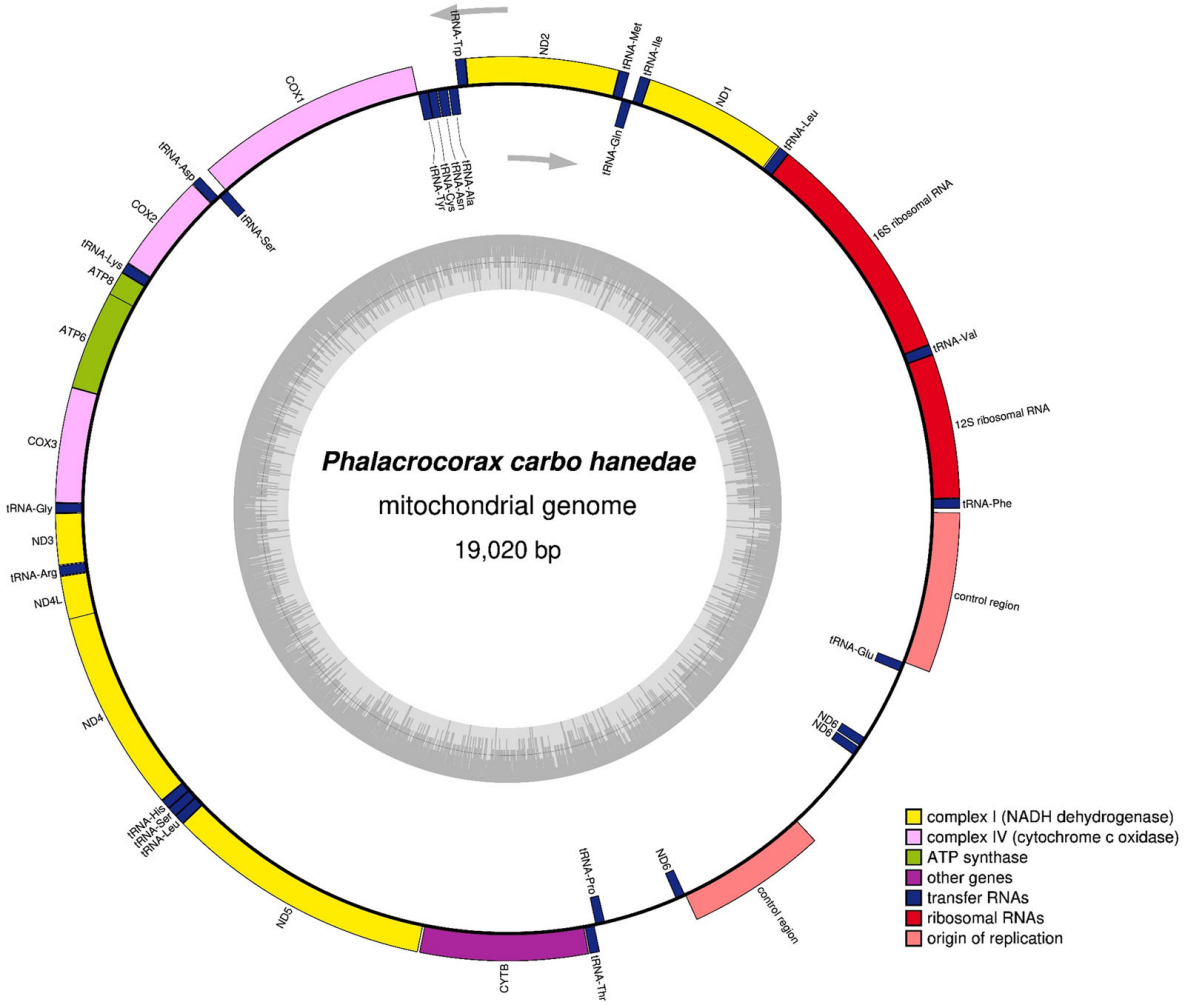
Gene map and organization of the complete mitochondrial genome of *Phalacrocorax carbo hanedae*, drawn by the OGDRAW version 1.3.1 (Grainer et al. 2019). Genes encoded on the heavy and light strand are shown outside and inside the circle, respectively. The inner grey ring indicates the GC content.

The complete mitogenome of *P. carbo hanedae* (DDBJ accession No. LC715365) was 19,020 bp in length, comprised 13 PCGs, two rRNA genes, 22 tRNA genes, and two control regions (Figure 2). Most mitochondrial genes were located on the heavy strand, except for *ND6* and eight tRNA genes (*trnQ*, *trnA*, *trnN*, *tnC*, *trnY*, *trnS*, *trnE*, and *trnP*). *P. hanedae* shares an identical gene arrangement with other Suliformes species, characterized by a duplicated region spanning the 3′ end of cytochrome *b* to the control region (Gibb et al. 2013). Its base composition was A (31.9%), C (31.9%), G (13.3%), and T (22.9%).

**Figure 3.**
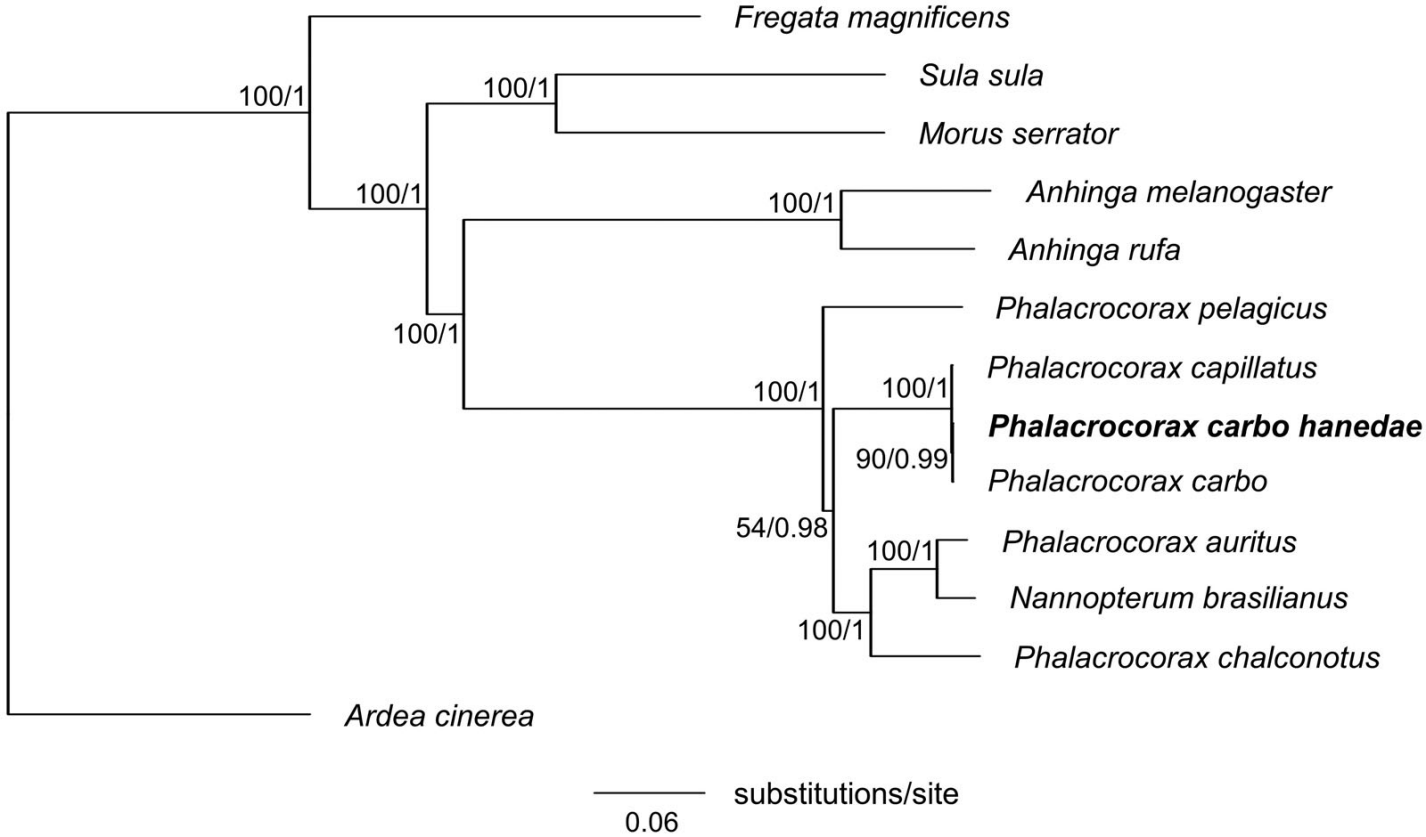
Maximum-likelihood tree of the order Suliformes based on the sequences of concatenated 13 protein-coding genes (PCGs) with the grey heron (*Ardea cinerea*) as an outgroup. Numbers beside each node indicate bootstrap support values for ML (left) and posterior probabilities support values for BI (right). The following sequences were used: *Anhinga melanogaster* MW042786 (Thomas et al. 2021), *Anhinga rufa* GU071055, *Ardea cinerea* KJ190947, *Fregata magnificens* MN356268, *Morus serrator* GU071056, *Nannopterum brasilianus* KT626611 (Rodrigues et al. 2017), *Phalacrocorax auratus* NC052822, *Phalacrocorax capillatus* LC714913 (Honda et al. 2022), *Phalacrocorax carbo* KR215630 (Zhang et al. 2017), *Phalacrocorax carbo hanedae* LC715365 (this study), *Phalacrocorax chalconotus* GU071054, *Phalacrocorax pelagicus* MN356399, and *Sula sula* LC541438.

Phylogenetic analysis based on BI and ML yielded identical phylogenetic trees (Figure 3). The overall topology of the order Suliformes was consistent with that previously reported (Gibb et al. 2013). We also confirmed the monophyly of *P. carbo* ssp. This study is expected to contribute to our understanding of the population genetic structure of *P. carbo hanedae* and the phylogenetic relationships among its subspecies.

## Supplementary Material

Supplemental Material

Supplemental Material

## Data Availability

The data that support the findings of this study are available in DDBJ at https://www.ddbj.nig.ac.jp/index-e.html under the accession no. LC715365. Gel image of long PCR products and Sanger sequencing results (ab1 files) are provided as electronic supplementary materials (Figure S1 and Data S1–S27).
