# Supplementary figures and images for: Complete mitochondrial genome of a subspecies of the great cormorant, Phalacrocorax carbo hanedae (Kuroda, 1925) (Suliformes: Phalacrocoracidae)
Source: Mitochondrial DNA B Resour. 2023 Jan 2;8(1):61–3. doi: 10.1080/23802359.2022.2160671 (PMC9815431; doi:10.1080/23802359.2022.2160671)

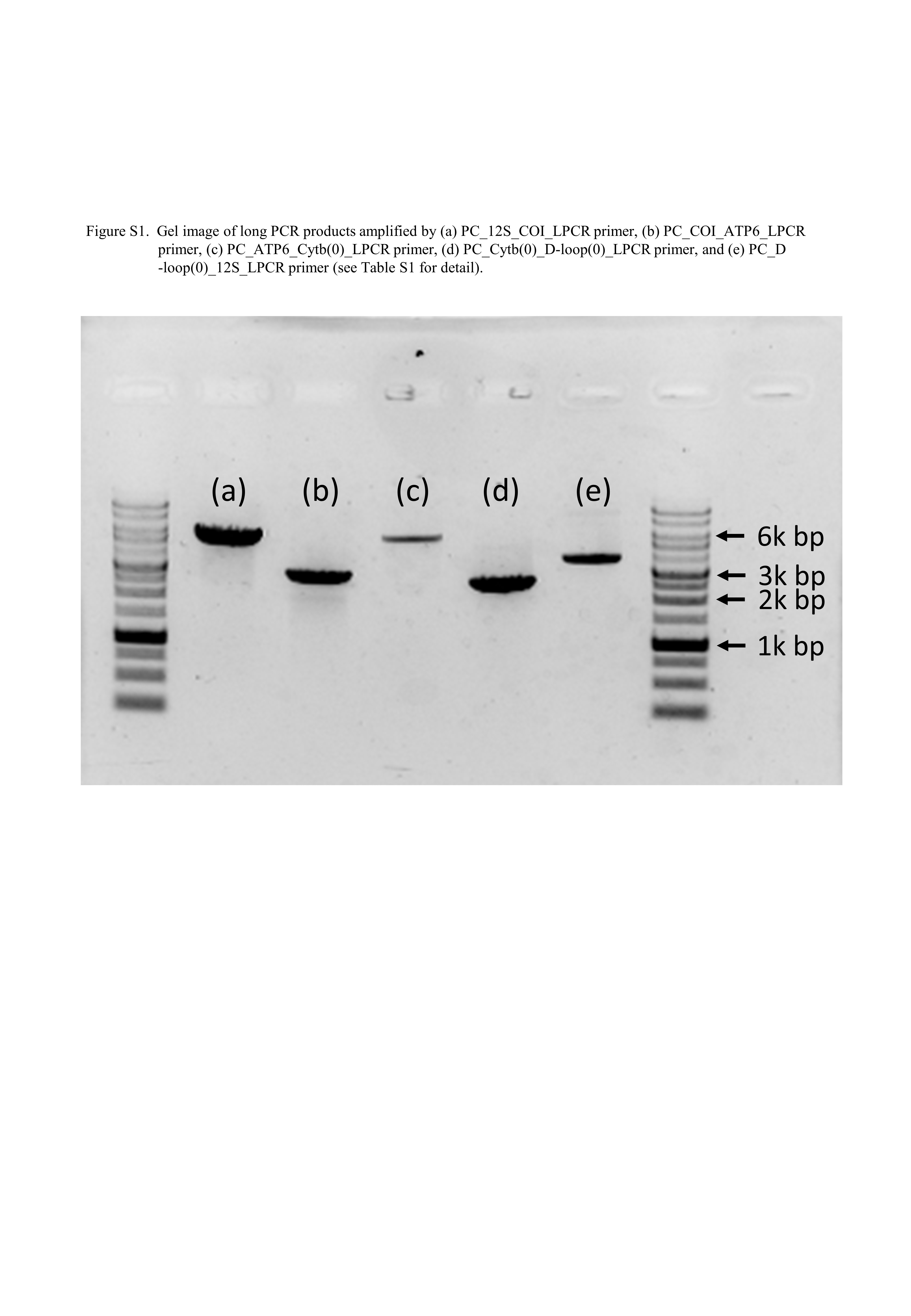

Supplement: Supplemental Material [file TMDN_A_2160671_SM3952.jpg]
