# Supplementary material for: Complete mitochondrial genome of a subspecies of the great cormorant, Phalacrocorax carbo hanedae (Kuroda, 1925) (Suliformes: Phalacrocoracidae)
Source: Mitochondrial DNA B Resour. 2023 Jan 2;8(1):61–3. doi: 10.1080/23802359.2022.2160671 (PMC9815431; doi:10.1080/23802359.2022.2160671)
Supplement: Supplemental Material [file TMDN_A_2160671_SM3951.docx]

Table S1. Primers used for the long PCR of mitochondrial DNA in *Phalacrocorax carbo hanedae*. Primer3* was used to develop the primers, using the mitogenome of *Phalacrocorax carbo* (Genbank accession No. KR21563) as the reference sequence.

| Primer name | Direction | Position (5’) | Primer sequence (5’ – 3’) | T_m_ (°C) |
| --- | --- | --- | --- | --- |
| PC_12S_COI_LPCR | Forward | 702‒ | AAACAGCCTACATACCGCCG | 57.6 |
|  | Reverse | ‒5887 | GATTGCCCCCAGGATTGAGG | 58.2 |
| PC_COI_ATP6_LPCR | Forward | 5566‒ | TGTAATTGTCACCGCCCACG | 58.0 |
|  | Reverse | ‒8500 | GTGAGGTTTGCTGTGAGTCG | 56.1 |
| PC_ATP6_Cytb(0)_LPCR | Forward | 8358‒ | CGAAACCAACCAACCATCTC | 54.0 |
|  | Reverse | ‒14254 | TGTGGTGAGACCTGCGATTA | 55.9 |
| PC_Cytb(0)_D-loop(0)_LPCR | Forward | 13926‒ | ATGGCGCATCATTCTTCTTC | 53.4 |
|  | Reverse | ‒16522 | TGGGTGGGTAGTGAAATTGG | 54.8 |
| PC_D-loop(0)_12S_LPCR | Forward | 16192‒ | GGCCTCTCCTCTTTTGGTTC | 55.6 |
|  | Reverse | ‒861 | TCACGTCCCCTTTCGTTAAG | 54.8 |

* Untergasser A, Cutcutache I, Koressaar T, Ye J, Faircloth BC, Remm M, Rozen SG (2012) Primer3 -new capabilities and interfaces. Nucleic Acids Research 40 (1): e115.

Table S2. Primers used for the sequencing of long PCR products in *Phalacrocorax carbo hanedae*.

| Primer name | Direction | Position (5’) | Primer sequence (5’ – 3’) |
| --- | --- | --- | --- |
| D-loop_12S_R7 | Reverse | ‒141 | TACACTGGGGCGCAGATACT |
| D-loop_12S_R6 | Reverse | ‒305 | TTGCTATGGCTAAGTCAAGTTTACA |
| D-loop_12S_R2 | Reverse | ‒579 | CACTTTAGTAGGGGAAGTGCCAAGA |
| 12S_NAD1_F1 | Forward | 1298‒ | AGCAAAGATAAACCCTTGTACCTCT |
| 12S_NAD1_F2 | Forward | 1962‒ | GGACCCGACTGTTTACCAAA |
| 12S_NAD1_F4 | Forward | 2540‒ | AATAGTCCTACGTGATCTGAGTTCA |
| NAD1_COI_F1 | Forward | 3339‒ | CCACTATGTCTCATCTTCTCATCCT |
| NAD1_COI_F2 | Forward | 4055‒ | CACAATCTCAAGCAACCATTG |
| 12S_COI_R2 | Reverse | ‒5523 | AGTTCTGCACGGATAAGTAGGCTGA |
| COI_ATP6_F2 | Forward | 6076‒ | GGGAGACCCAGTCCTATACCAACAC |
| COI_ATP6_F3 | Forward | 6600‒ | TCACAGGTACACCCTCCAC |
| COI_ATP6_F4 | Forward | 7279‒ | AACAATCCTACCAGCCATCG |
| ATP6_D-loop_F2 | Forward | 8875‒ | AGAAAGCACATTCCAAGGACATCAC |
| ATP6_D-loop_F3 | Forward | 9397‒ | CGCAGCCTGATATTGACACT |
| ATP6_NAD5_F1 | Forward | 9935‒ | GACCATAGCCTGACACTATGACTTT |
| ATP6_NAD5_F2 | Forward | 10663‒ | GAAACCAACCAGAACGCCTA |
| ATP6_NAD5_F6 | Forward | 11340‒ | AACCTCACAAACATAGCACTTCC |
| ATP6_NAD5_R2 | Reverse | ‒12522 | ATTGGGCAGATTTTCCTGTG |
| ATP6_NAD5_R3 | Reverse | ‒12712 | GTAGCAGCGAATAGGGTGGA |
| NAD5_D-loop_F1 | Forward | 12594‒ | ACAATAGTTGTTGCTGGAATCTTTC |
| NAD5_D-loop_F2 | Forward | 13276‒ | CCCCCATAACCATACCCCTA |
| CYTB_NAD6_F1 | Forward | 14432‒ | GAAAACTTCACCCCAGCAA |
| NAD6(0)_D-loop(0)_F | Forward | 15185‒ | AAACAATCCTCCACCATCCA |
| D-loop_12S_F2 | Forward | 16194‒ | CCTCTCCTCTTTTGGTTCCCTTTTT |
| D-loop_12S_F3 | Forward | 16730‒ | GCCCTATTTTCCCCAAACCT |
| D-loop_12S_F4 | Forward | 17599‒ | ATCAGCAGCCAAAGACACAGAATA |
| D-loop_12S_F6 | Forward | 18224‒ | CATTAGTGGACATGGAATGGTTT |
